# Protective Effects of Recombinant *Lactobacillus paracasei* Expressing Porcine β-Defensin 2 Against DSS-Induced Colitis in a Murine Model

**DOI:** 10.3390/ani16101425

**Published:** 2026-05-07

**Authors:** Ying Chen, Zhixuan Guo, Fangjie Yin, Yiting Guo, Jiaxuan Li, Xiaona Wang

**Affiliations:** 1 College of Veterinary Medicine, Northeast Agricultural University, Harbin 150030, China; 15662788212@163.com (Y.C.); 15776883739@163.com (Z.G.); 18754378593@163.com (F.Y.); ytingguo@126.com (Y.G.); 2 Heilongjiang Key Laboratory for Animal Disease Control and Pharmaceutical Development, Harbin 150030, China

**Keywords:** *Lactobacillus paracasei*, porcine β-defensin-2, inflammatory bowel disease, intestinal mucosal injury repair

## Abstract

Porcine β-defensin 2 (pBD2) possesses broad-spectrum antimicrobial properties and is crucial for gastrointestinal mucosal repair. Lactic acid bacteria (LAB) serve as optimal vectors for exogenous protein delivery due to their high biosafety, intestinal colonization capacity, and ability to modulate gut microecology. In this study, we engineered a recombinant *Lactobacillus paracasei* strain (pPG-N1-pBD2/*27-2*) that efficiently secretes pBD2. In vitro, this recombinant strain significantly enhanced the proliferation and migration of porcine intestinal epithelial cells (IPEC-J2). In vivo, oral administration of pPG-N1-pBD2/*27-2* markedly alleviated dextran sulfate sodium (DSS)-induced colitis in mice. This protective effect was evidenced by reduced Disease Activity Index (DAI) scores, prevention of colon shortening, and decreased colonic activities of myeloperoxidase (MPO) and eosinophil peroxidase (EPO), alongside normalized N-acetyl-β-D-glucosaminidase (NAG) levels. Histopathological analysis revealed that the treatment preserved mucosal architecture, including goblet cells and crypts, and fortified the physical barrier by upregulating tight junction proteins. Mechanistically, the recombinant strain suppressed the colonic iNOS/COX-2 inflammatory axis, decreased serum pro-inflammatory cytokines (IL-6, IL-1β, and TNF-α), and elevated the anti-inflammatory cytokine IL-10. Furthermore, it restored systemic immune homeostasis by normalizing the proportions of splenic macrophages, T/B lymphocytes, and natural killer (NK) cells. In conclusion, pPG-N1-pBD2/*27-2* mitigates colitis through a dual mechanism: reinforcing the intestinal physical barrier and rebalancing the innate–adaptive immune axis. These findings highlight the potential of pBD2-engineered probiotics as novel biological therapeutics for intestinal inflammatory diseases.

## Simple Summary

Inflammatory bowel diseases are a major challenge in both human and animal medicine, necessitating the development of safe and effective biological treatments. This study engineered a probiotic strain, *Lactobacillus paracasei*, to produce and secrete a natural defense protein called porcine β-defensin 2 (pBD2). Using a mouse model of intestinal inflammation, we found that this engineered probiotic significantly reduced clinical symptoms and prevented gut damage. Our results show that the probiotic works by both physically strengthening the intestinal wall and balancing the immune system’s response to injury. This research suggests that using beneficial bacteria to deliver natural defense proteins is a promising strategy for treating intestinal diseases and improving gut health in animals.

## 1. Introduction

Dysbiosis of the gut microbiota and compromised intestinal barrier integrity are hallmarks of inflammatory bowel disease (IBD), a chronic, recurrent illness [1]. Clinically, there are two primary subtypes of IBD: ulcerative colitis (UC) and Crohn’s disease (CD) [2,3]. Rectal bleeding, diarrhea, and abdominal pain are symptoms of IBD. In recent years, mucosal healing has emerged as a vital therapeutic endpoint in the management of IBD, shifting the clinical emphasis from mere symptom control to the restoration of intestinal epithelial integrity.

Defensins serve as crucial immunomodulators that effectively connect innate and adaptive immunity [4]. Their regulation is closely linked to the etiology of autoimmune and inflammatory diseases. Clinically, patients diagnosed with Crohn’s disease frequently display reduced levels of ileal α-defensin and colonic β-defensin, highlighting their essential role in preserving epithelial tight junctions and maintaining mucosal barrier integrity [5]. Koeninger et al. demonstrated that HBD2 significantly enhance the DAI and DSS models in mice, effectively preventing colitis, which is characterized by shortened colon length and weight loss [6]. In contrast, mice lacking matrix metalloproteinase-7 (MMP7), an enzyme critical for the maturation of defensins, exhibit heightened susceptibility to DSS-induced colitis and increased colonic IL-1β levels [7]. However, several challenges hinder the practical application of β-defensins. One major limitation is that their chemical synthesis is technically demanding, resulting in low production efficiency and high manufacturing costs. Therefore, the development of genetically engineered probiotics as live delivery vectors for the expression of exogenous defensins has emerged as a pressing and promising strategy.

Lactic acid bacteria (LAB) can alleviate intestinal dysbiosis in patients with IBD by competing with harmful microorganisms and promoting the growth and proliferation of beneficial microbes [8]. Owing to their biological characteristics and their essential role in maintaining intestinal homeostasis, LAB can act synergistically with defensins to protect intestinal barrier integrity and immune balance [9]. Belo GA et al. [10] demonstrated that the S-layer protein from *Lactococcus*, when combined with *Lactococcus* NCDO 2118, alleviates colitis symptoms in mice induced by DSS. The extracellular vesicles secreted by this combination can downregulate proliferative cytokines, thereby improving colitis in affected mice [11]. Furthermore, recombinant *Lactobacillus* is delivery system for bioactive substances, such as cytokines and antimicrobial peptides, enabling targeted modulation of mucosal immune responses and significantly augmenting host intestinal defenses. For example, the recombinant *Lactobacillus* engineered by Qiu et al. [12] to express anti-inflammatory cytokine IL-10 markedly improves the pathological damage associated with colitis caused by Streptococcus by inhibiting the NF-κB pathway. Consequently, *Lactobacillus* can function as effective oral mucosal carriers. Utilizing these bacterias to express defensins not only addresses the challenges associated with the production and application of defensins but also amplifies the probiotic effects of *Lactobacillus*. Due to its exceptional ability for intestinal colonization and resistance to stress, *L. paracasei 27-2* is an excellent vector for the expression of exogenous proteins [13].

In this study, porcine-derived *L. paracasei 27-2* was genetically engineered to achieve efficient expression of pBD2. It extensively investigated the biological activity of the recombinant strains and evaluated its reparative effects on intestinal injury in murine colitis models. This selection guarantees compatibility between the antimicrobial peptide and its bacterial chassis, while capitalizing on the highly conserved anti-inflammatory and mucosal protective functions of β-defensins across mammalian species. Moreover, this design enhances the potential application of the engineered probiotic in the management of porcine intestinal health.

## 2. Materials and Methods

### 2.1. Bacterial Strain and Plasmid

The growth of *Lactobacillus paracasei 27-2* (*L. paracasei 27-2*) occurred in de Man Rogosa and Sharpe (MRS) broth at 37 °C. The expression plasmid pPG-T7g10-PPT (pPG-PPT) was synthesized in our laboratory.

### 2.2. Construction of Recombinant Strain

In this study, a highly efficient native regulatory sequence, *N1*, derived from *L. paracasei 27-2* and previously characterized in our laboratory, was employed to enhance the pPG-PPT expression system. The porcine β-defensin 2 (*pBD2*) gene was amplified by PCR from freshly isolated porcine liver genomic DNA. The *pBD2* fragment was fused with the *N1* regulatory sequence using overlap extension PCR, and the resulting construct was inserted into the pPG-PPT vector to generate pPG-N1-pBD2. Electroporation of *L. paracasei 27-2* with the recombinant plasmid generated the recombinant strain designated pPG-N1-pBD2/*27-2*. A His-tag was incorporated into the *N1-pBD2* fusion protein to facilitate detection.

### 2.3. Protein Expression

The pPG-N1-pBD2/*27-2* and pPG-PPT/*27-2* were cultured to an OD_600nm_ of 1.0. After centrifugation, the pellets were treated with lysozyme, washed, resuspended in phosphate-buffered saline (PBS), and sonicated. Supernatant proteins were concentrated using TCA-acetone precipitation. Following SDS-PAGE resolution, the samples were transferred to PVDF membranes and incubated with mouse anti-His monoclonal antibody (1:1000, Abmart, Shanghai, China), followed by HRP-conjugated goat anti-mouse IgG (1:5000, Sigma, Ronkonkoma, NY, USA). Protein bands were detected through the use of enhanced chemiluminescence (ECL, Thermo Scientific, Durham, NC, USA).

To confirm protein expression and localization, indirect immunofluorescence (IFA) was conducted. The technique is delineated as follows: An overnight culture of recombinant strains was cultivated for 16 h in MRS broth with chloramphenicol (10 µg mL^−1^), followed by centrifugation at 12,000× *g* for 5 min. The mouse anti-flag monoclonal antibody (Abmart, Shanghai, China) was used at a dilution of 1:2000, in conjunction with fluorescein isothiocyanate (FITC)-conjugated goat anti-mouse IgG (Invitrogen, Carlsbad, CA, USA) diluted at 1:4500, and incubated at ambient temperature for 3 h. Following this, the samples were washed three times with sterile PBS and then incubated with 4′,6′-diamino-2-phenylindole (DAPI) (Invitrogen, Carlsbad, CA, USA) for 5 min. Afterward, the samples were washed three times with sterile PBS, dehydrated with water, and analyzed using a fluorescence microscope (Zeiss, Oberkochen, Germany).

The expression levels of pBD2 in bacterial precipitation and culture supernatants were assessed at various time points (6, 10, 14, 18, 22, and 24 h) using an indirect enzyme-linked immunosorbent assay (ELISA). Briefly, 96-well plates received a coating of 100 μL from each sample and were incubated overnight at 4 °C. After being cleaned with PBST, the plates were blocked with 5% skim milk (2 h, 37 °C). Subsequently, a mouse anti-His monoclonal antibody was added to the plates. Immunoreactivity was visualized using TMB substrate. The OD_450nm_ value was measured, and protein concentrations were determined based on a standard curve.

### 2.4. Biological Characterization of the pPG-N1-pBD2/27-2

The pPG-N1-pBD2/*27-2* and pPG-PPT/*27-2* were inoculated into MRS and cultured for 24 h. Growth kinetics were monitored by determining viable cell counts every 2 h using the standard plate count method. To assess plasmid segregational stability, the pPG-N1-pBD2/*27-2* strain underwent serial passage for 20 generations. Plasmid DNA and total protein were extracted at 5-generation intervals up to the 20th generation. Plasmid retention was confirmed by PCR employing primers pPG-F/R, while expression stability was evaluated through analysis of the extracted proteins.

The pPG-PPT/*27-2* and pPG-N1-pBD2/*27-2* were cultured under acidic (pH 2–5) and bile salt (0.1% and 0.5%) conditions. Following incubation, samples were serially diluted tenfold and plated for colony enumeration following an additional 24 h incubation at 37 °C. Each treatment was performed in triplicate. Survival rates under acid and bile salt stress were calculated as follows: survival rate (%) = (viable cell count of the treated group/viable cell count of the control group) × 100%.

### 2.5. Analysis of the Activity of pBD2 In Vitro

The pPG-N1-pBD2/*27-2* was inoculated into MRS at a 1:100 dilution (*v*/*v*) and cultured. Culture supernatants were collected, and proteins were precipitated through stepwise ammonium sulfate saturation at 20%, 40%, and 60% concentrations at 0 °C. Following each step, samples were centrifuged (12,000 rpm, 15 min). The precipitates were then dissolved in PBS and dialyzed against PBS for 48 h, with buffer replacement occurring every 6 h. After dialysis, the protein solutions were stored at −80 °C. The resulting partially concentrated protein preparation was directly used in subsequent in vitro assays. This approach is consistent with the study design, as *L. paracasei* exerts its therapeutic effect in vivo via viable bacteria secreting target peptides, rather than as isolated pure protein.

The cytotoxicity and proliferative effects of pBD2 on IPEC-J2 cells were assessed using the CCK-8 assay. In 96-well plates, cells were cultivated and allowed to incubate until they achieved around 70% confluence. To evaluate cytotoxicity, the pBD2 protein was administered to cells for 12 h at final concentrations varying from 0 to approximately 200 μg/mL. For proliferation analysis, cells were exposed to pBD2 at concentrations between 12.5 and 200 ng/mL for 12 h. Following treatment, 10 μL of CCK-8 reagent was introduced to each well, and the plates were kept in the dark for incubation. Cell viability and proliferation capacity were determined according to the OD_450nm_ absorbance values. Negative controls and multiple replicate wells were included for each treatment. All in vitro cell-based assays were performed with 3 independent biological repeats.

The purpose of the cell scratch test was to evaluate how pBD2 affected IPEC-J2 cells’ ability to migrate [14]. IPEC-J2 cells were trypsinized, resuspended in full media, and seeded into 6-well plates with 100 μL of sterile ibidi inserts when they reached confluence. The cells were incubated until they reached approximately 80% confluence, at which point the inserts were carefully removed. Subsequently, pBD2 at the optimal concentration was added to stimulate the IPEC-J2. The healing area of the scratch was observed under an inverted fluorescence microscope at 0 h, 12 h, and 24 h.

### 2.6. Animal Model

**Figure 1:**
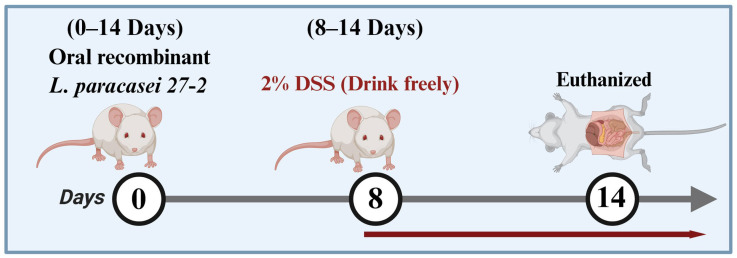
DSS-induced colitis model. From day 0 to day 14, mice were orally administered with recombinant *L. paracasei 27-2*. On day 8, colitis was induced by providing unrestricted access to 2% (*w*/*v*) dextran sulfate sodium (DSS) solution (highlighted in red) for 7 days, with the solution refreshed every 2 days. All mice were euthanized on day 14.

This study used thirty-two BALB/c mice that were six weeks old. Male mice were used in this animal experiment, and mice were randomly allocated into different groups. Blinded assessment was performed for histopathological analysis. The mice were divided randomly into four groups, with each group containing eight mice and housed separately in cages. The individuals were acclimated to a controlled environment with a temperature of 22 ± 2 °C, consisting of 12 h of light and 12 h of dark. Mice were orally administered either the pPG-N1-pBD2/*27-2* or the control strain pPG-PPT/*27-2* daily for 14 consecutive days. On day 8, colitis was induced by providing unrestricted access to a 2% (*w*/*v*) dextran sulfate sodium (DSS) solution for 7 days; the DSS solution was refreshed every 2 days [15,16,17]. All mice were euthanized at the end of study period. Experimental groupings and administration dosages are detailed in Figure 1 and Table 1. Animals within each group (*n* = 8) were prospectively allocated to predefined experimental endpoints to ensure adequate sample collection for different analyses. All mice were included for sample collection and subsequent individual parameter detection and statistical analysis. No animals or data were excluded from the analyses. Body weight, overall health, and the existence of diarrhea were tracked every day during the DSS induction period. As directed by the manufacturer, a Pyramidon-based kit was used to collect fecal samples in order to detect occult blood. The scoring criteria listed in Table 2 were used to calculate the DAI [18]. The length of the complete colonic segment, which ran from the cecum to the anus, was measured after euthanasia.

### 2.7. H&E Staining

A Skiving Machine Slicer was used to cut fresh colon samples into 5 μm sections after they had been fixed in 4% paraformaldehyde for the entire night, rinsed with running water for two hours, rehydrated with gradient ethanol, and embedded in paraffin. Hematoxylin and eosin (H&E) staining was applied to the sections in accordance with normal protocol. A digital section scanner was used to take pictures of the colonic morphology [16].

### 2.8. Analysis of Tight Junction Protein Expression in the Colon

Western blotting and real-time quantitative RT-PCR (qRT-PCR) were used to assess the amounts of protein and mRNA expression in colon tissues, respectively; freezing immunofluorescence staining was used to assess the localization of proteins.

For Western blot analysis, the colon tissues were broken down in ice-cold RIPA lysis buffer that included protease and phosphatase inhibitors. The supernatant was collected after centrifugation (12,000 rpm, 4 °C, 15 min). Following SDS-PAGE resolution, the samples were transferred to PVDF membranes. The membranes were incubated with Claudin-2, ZO-1, and Occludin monoclonal antibody (1:500, Servicebio, Wuhan, China), followed by HRP-conjugated goat anti-mouse IgG (1:5000).

For the qRT-PCR analysis, total RNA was extracted from colon tissues, and cDNAs were generated using a reverse transcription reagent kit. Following cDNA synthesis via reverse transcription, the mRNA levels of ZO-1, Claudin-2, and Occludin were quantified. The qPCR value was calculated using the 2^−ΔΔct^ method and normalized to β-actin. Primers used for qPCR are given in Table S1.

Frozen tissue samples embedded in OCT were sectioned (5 μm), air-dried, and fixed in cold acetone at 4 °C [19]. Following a 16 h incubation at 4 °C with primary antibodies against ZO-1 (Servicebio, GB151981, 1:100 dilution), Claudin-2 (Invitrogen, Product # 325600, 1:100 dilution), and Occludin (Servicebio, GB111401, 1:100 dilution), the sections were exposed to CY3-conjugated secondary antibodies (Servicebio, CY3-conjugated Goat Anti-Rabbit IgG, GB21303, CY3-conjugated Goat Anti-Mouse IgG, GB21301, 1:300 dilution) for two hours in the dark. DAPI was used to counterstain the cell nuclei, and a confocal laser scanning microscope was used to take pictures.

### 2.9. Effects of Recombinant Microorganisms on the Levels of Colitis-Associated Enzymes

EPO, iNOS, and COX-2 concentrations were measured, together with the activity of the immune cell marker enzymes MPO and NAG in colonic tissues. Briefly, colonic tissues were collected and weighed, and a 5% (*w*/*v*) tissue homogenate was prepared by homogenizing the samples in homogenization buffer at a tissue-to-buffer ratio of 1:19 (*w*/*v*). The tissues were homogenized thoroughly to ensure uniformity. All measurements were then performed according to the manufacturers’ instructions of the respective assay kits (Jiangsu Meimian Industrial Co., Ltd., Yancheng, China), and standard curves were generated for quantitative analysis. All assays were completed within 24 h after sample preparation to avoid repeated freeze–thaw cycles that could affect protein activity.

### 2.10. Cytokine Detection

ELISA was used to determine the cytokine levels in mouse serum. Serum samples were collected on day 14 after successful establishment of DSS-induced colitis model. TNF-α, IL-1β, IL-6, and IL-10 levels were measured in accordance with the manufacturers’ instructions.

### 2.11. Analysis of Immune Cell Populations in the Spleen

Mice were euthanized, and spleens were aseptically collected under a biosafety cabinet. The spleens were placed on a sterile 200-mesh nylon screen, and 3 mL of lymphocyte separation medium was added. The tissues were gently dissociated using the plunger of a 10 mL syringe within 3 min. After passing through a 70 μm cell strainer, the cell suspension was gathered into 15 mL sterile centrifuge tubes. Subsequently, 3 mL of RPMI-1640 medium was slowly added, followed by centrifugation (1000 rpm, 25 min). The cells were resuspended in sterile PBS after being treated for two minutes with red blood cell lysis buffer. Cell numbers were adjusted to 1 × 10^6^ cells/mL.

APC-conjugated anti-mouse CD3e, PE-conjugated anti-mouse CD49b, FITC-conjugated anti-mouse CD45R, and PE-conjugated anti-mouse F4/80 monoclonal antibodies (Invitrogen, USA) were used to stain the cells for 30 min at 37 °C in the absence of light. Cells were cleaned, then resuspended in PBS and subjected to BD FACSCalibur analysis.

### 2.12. Statistical Analysis

The data are expressed as the mean ± standard deviation (SD) of three replicates and were analyzed using GraphPad Prism version 10.1.2. To assess the significance between the treatment and control groups, Tukey’s multiple comparison test was performed after a one-way analysis of variance (ANOVA). A *p*-value below 0.05 was considered statistically significant. All experiments were performed in at least three independent biological replicates to ensure reproducibility.

## 3. Results

### 3.1. Construction of pPG-N1-pBD2/27-2 and Detection of pBD2 Expression

**Figure 2:**
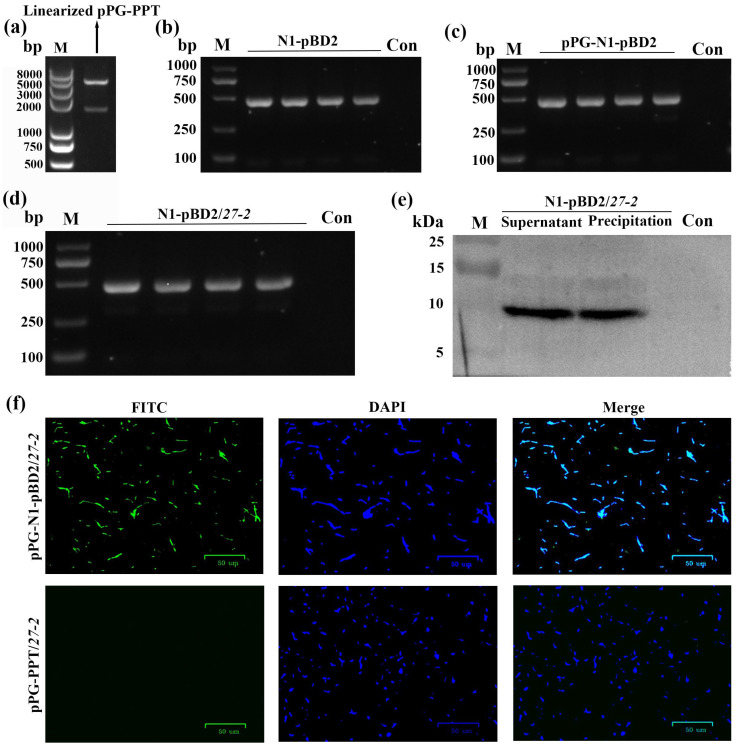
The pPG-N1-pBD2/*27-2* expressing pBD2 were identified. (**a**). Double enzyme digestion results of pPG-PPT plasmid. (**b**) PCR amplification of the *N1-pBD2* fragment. (**c**) PCR identification of the pPG-N1-pBD2 plasmid. (**d**) PCR Identification results of pPG-N1-pBD2/*27-2*. (**e**) Western blot identification of the supernatant and precipitation from pPG-N1-pBD2/*27-2* culture. (**f**) Identification of proteins expressed in the pPG-N1-pBD2/*27-2*. Mouse anti-flag monoclonal antibody was used as the primary antibody, and goat anti-mouse IgG labeled with FITC was used as the secondary antibody (green). Subsequently, the cell nuclei were stained with DAPI (blue), and the results were observed under an inverted fluorescence microscope. Scale bar = 50 μm.

The high-efficiency regulatory sequence *N1* and the *pBD2* gene were fused by overlap PCR (Figure 2b), and the resulting *N1–pBD2* fragment was ligated into the linearized pPG-PPT plasmid (Figure 2a) to generate the recombinant plasmid pPG-N1-pBD2 (Figure 2c). The recombinant plasmid was subsequently introduced into *L. paracasei 27-2* by transformation and obtained positive transformants. As shown in Figure 2d, PCR was used to confirm the fusion fragment *N1-pBD2* (481 bp) from single colonies. The pBD2 protein in both the culture supernatant and cell precipitate of pPG-N1-pBD2/27-2 were detected (Figure 2e). In addition, the expression of pBD2 in pPG-N1-pBD2/*27-2* was further verified by indirect IFA (Figure 2f). The pPG-N1-pBD2/*27-2* exhibited a typical short rod-shaped morphology accompanied by distinct green fluorescence, whereas no specific green fluorescence was observed in the pPG-PPT/*27-2*.

The protein expression levels of pPG-N1-pBD2/*27-2* cultured for different time periods were determined using an indirect ELISA assay. The protein concentrations of each pPG-N1-pBD2/*27-2* were calculated based on a standard curve (Table 3). The results indicated that after 18 h of cultivation, the expression levels of pBD2 protein in both the precipitation and the culture supernatant reached their maximum values. Specifically, the maximum expression level of pBD2 in the supernatant was 0.64 μg/mL, while that in the bacterial cells was 2.21 μg/mL.

### 3.2. Biological Characterization of pPG-N1-pBD2/27-2

**Figure 3:**
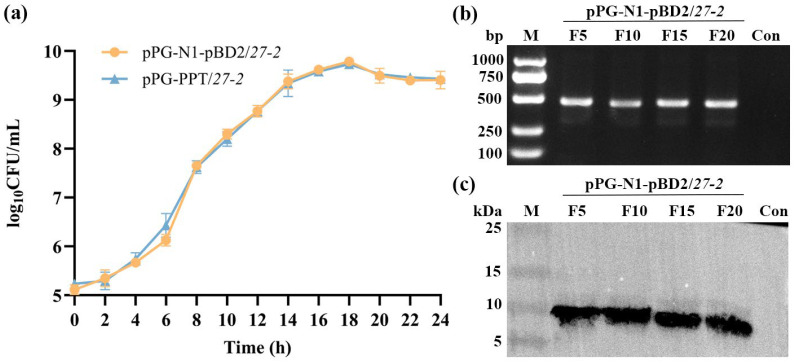
The stability and growth properties of pPG-N1-pBD2/*27-2* were examined. (**a**) The determination of growth curve of pPG-N1-pBD2/*27-2*. (**b**,**c**) The genetic and protein expression stability of pPG-N1-pBD2/*27-2*.

To investigate the growth characteristics of the pPG-N1-pBD2/*27-2*, a growth curve assay was conducted. At around 4 h, the pPG-N1-pBD2/*27-2* entered the logarithmic growth phase, and at about 16 h, it reached the stationary phase. The growth curve exhibited a typical sigmoidal (S-shaped) pattern, which was consistent with the growth trend of the parental strain pPG-PPT/*27-2* (Figure 3a). The results indicated that, even after 20 generations of continuous passage, both the 481 bp *N1-pBD2* fragment and the 10 kDa pBD2 protein were consistently detected (Figure 3b,c). These findings align with expectations and confirm the stability of plasmid maintenance and protein expression in the pPG-N1-pBD2/*27-2*.

The acid and bile salt tolerance of the recombinant strain pPG-N1-pBD2/*27-2* and the parental strain pPG-PPT/*27-2* was assessed using the viable plate count method. As indicated in Table 4 and Table 5, both strains demonstrated tolerance to acidic conditions (pH 2–5) and bile salts (0.1% and 0.5%). Although the number of viable cells decreased with lower pH and higher bile salt concentrations, no significant differences were found between the recombinant and parental strains under the same conditions.

### 3.3. Analysis of the In Vitro Activity of pBD2 Protein Expressed by pPG-N1-pBD2/27-2

**Figure 4:**
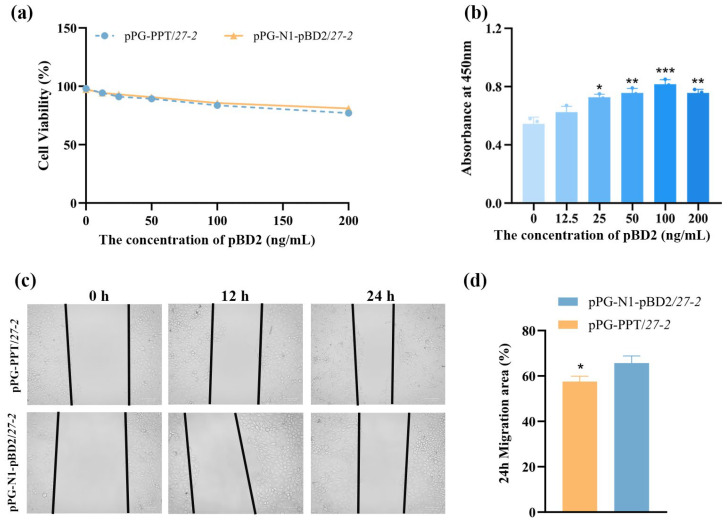
Evaluation of pBD2 activity in vitro. The toxicity assessment (**a**) and proliferation assay (**b**) of pBD2 protein expressed by pPG-N1-pBD2/*27-2* on IPEC-J2 were analyzed. (**c**,**d**) Using the optimal concentrations of each protein for cell proliferation, the above-mentioned proteins were added, respectively, after the cell scratch assay. The healing area of the scratch was observed under an inverted fluorescence microscope at 0 h, 12 h, and 24 h. (* *p* < 0.05, ** *p* < 0.01, *** *p* < 0.001).

The effects of pBD2 on IPEC-J2 viability and proliferation were evaluated using a CCK-8 assay. Results indicated that the recombinant protein was non-cytotoxic across the tested concentration range (12.5–200 ng/mL) (Figure 4a). Moreover, pBD2 significantly promoted cell proliferation, with the maximal effect observed at 100 ng/mL (Figure 4b). The experiment was performed using the optimal protein concentration determined by the CCK-8 assay described above. After co-incubation of pBD2 protein with IPEC-J2 for 12 h, cells at the wound edges began to migrate toward the center of the scratch, indicating a clear wound-healing trend (Figure 4c). Quantitative analysis of the migrated area at 24 h using ImageJ 1.54 g demonstrated that, compared with the pPG-PPT/*27-2*, the pBD2-treated group exhibited a significantly increased cell migration area (*p* < 0.05; Figure 4d). The pBD2 protein effectively accelerates the repair of scratch-induced injury by significantly enhancing the migratory activity of IPEC-J2.

### 3.4. Effect of pPG-N1-pBD2/27-2 on DSS-Induced Colitis in Mice

**Figure 5:**
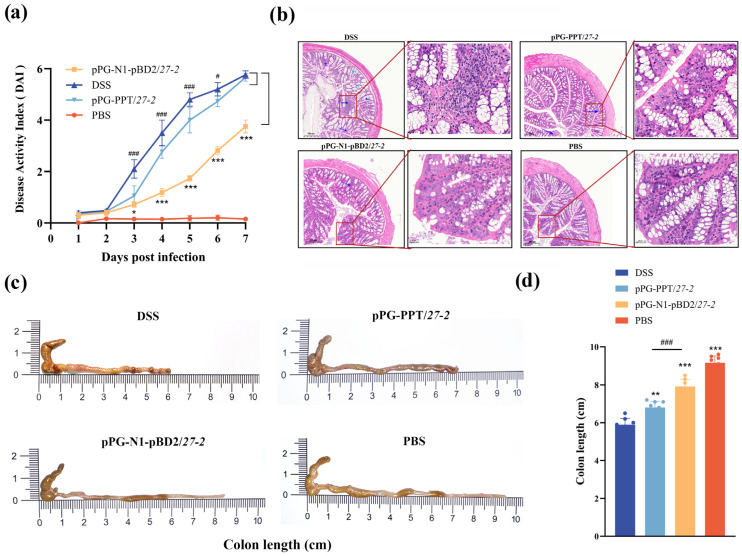
The orally administered recombinant strain pPG-N1-pBD2/*27-2* exerted protective effects against intestinal injury in a mouse model of colitis. (**a**) DAI scores of mice in each group. (**b**) H&E staining. Note: inflammatory cell infiltration is indicated by dark blue arrows; light blue arrows indicate morphologically damaged goblet cells. (**c**,**d**) Each mouse group’s colon length was measured. (* *p* < 0.05, ** 0.01 < *p* < 0.05, *** *p* < 0.01 vs. DSS; ^#^ *p* < 0.05, ^###^ *p* < 0.01 vs. pPG-N1-pBD2/*27-2*; *n* = 3, per group). DAI data were analyzed by repeated-measures ANOVA.

The DSS group displayed typical symptoms of colitis, including weight loss, loose stools, and fecal occult blood, beginning on day 3. This resulted in a significant, time-dependent increase in DAI scores (*p* < 0.01). Although pPG-PPT/*27-2* offered partial relief, pPG-N1-pBD2/*27-2* exhibited superior efficacy, as evidenced by significantly lower DAI scores compared to both the DSS and pPG-PPT/*27-2* groups (*p* < 0.01; Figure 5a).

H&E staining indicated that the PBS control group maintained intact intestinal mucosal architecture, regular crypt architecture, minimal inflammatory infiltration, and normal morphological integrity of goblet cells. Conversely, the DSS-induced colitis group displayed severe epithelial damage, robust inflammatory cell infiltration, disrupted crypt architecture and extensive crypt injury, accompanied by obvious morphological damage of goblet cells. Although the pPG-PPT/*27-2* group showed mild protective effects, the pPG-N1-pBD2/*27-2* group exhibited markedly improved mucosal epithelial integrity, reduced inflammatory infiltration, restored crypt structure and relieved goblet cell morphological injury (Figure 5b).

Colon shortening is a characteristic feature of DSS-induced colitis. In comparison to the PBS group, the DSS group demonstrated significant colon shortening. However, oral administration of pPG-N1-pBD2/*27-2* mitigated this effect to varying extents (Figure 5c). While treatment with pPG-PPT/*27-2* resulted in a partial improvement in colon length (*p* < 0.05), the pPG-N1-pBD2/*27-2* group exhibited a significantly greater recovery (*p* < 0.01). These results underscore the enhanced protective efficacy of pPG-N1-pBD2/*27-2* against DSS-induced macroscopic damage (Figure 5d).

### 3.5. Regulatory Effects of pPG-N1-pBD2/27-2 on the Tight Junction Proteins

**Figure 6:**
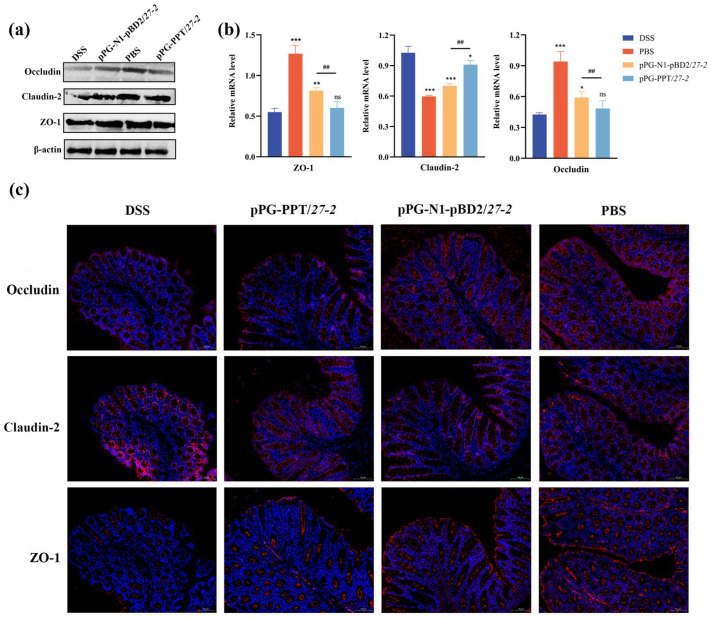
Measurement results of colonic tight junction protein content in each group of mice. TJP expression in the mouse colon as determined by Western blot (**a**), qPCR (**b**), and frozen tissue immunofluorescence (**c**). The blue fluorescence (DAPI) marks the cell nucleus, and red fluorescence (Cy3) marks the tight junction proteins. (* *p* < 0.05, ** 0.01 < *p* < 0.05, *** *p* < 0.01 vs. DSS; ^##^ 0.01 < *p* < 0.05 vs. pPG-N1-pBD2/*27-2*; *n* = 3, per group, ns: not significant (*p* ≥ 0.05)).

Western blot and qRT-PCR analyses showed that DSS treatment significantly reduced the expression of barrier-forming tight junction proteins, including ZO-1 and Occludin, compared with the PBS control group (*p* < 0.01; Figure 6a,b). In contrast, treatment with pPG-N1-pBD2/*27-2* markedly restored their expression levels, which were significantly higher than those in both the DSS and pPG-PPT/*27-2* groups (*p* < 0.05; Figure 6a,b). Notably, Claudin-2 exhibited an opposite trend. Its expression was significantly increased in the DSS group compared with the PBS control (*p* < 0.01), whereas treatment with pPG-N1-pBD2/*27-2* effectively reduced its expression (*p* < 0.05; Figure 6a,b). Immunofluorescence analysis of frozen sections (Figure 6c) showed that the expression patterns of colonic tight junction proteins across all groups were consistent with the results described above. These results indicate that pPG-N1-pBD2/27-2 enhances epithelial barrier integrity by upregulating barrier-forming proteins (ZO-1 and Occludin) while suppressing the pore-forming protein Claudin-2.

### 3.6. Effects of pPG-N1-pBD2/27-2 on Colitis-Associated Enzyme Activity and Inflammatory Cytokine Levels

**Figure 7:**
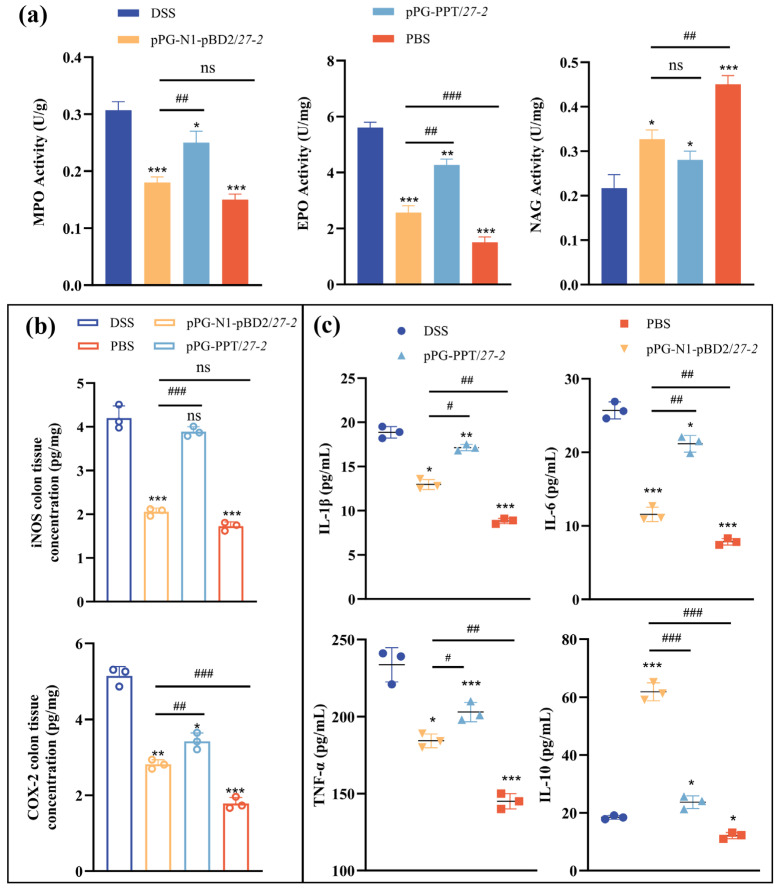
Effects of pPG-N1-pBD2/*27-2* on colitis-related enzyme levels in the colon and serum cytokine levels in mice. (**a**) The activity of MPO, EPO, and NAG in mouse colons across treatment groups. (**b**) Measurement results of iNOS and COX-2 related enzyme concentrations in each group of mice. (**c**) Detection results of inflammatory cytokine secretion levels in mice of each group. (* *p* < 0.05, ** 0.01 < *p* < 0.05, *** *p* < 0.01 vs. DSS; (^#^ *p* < 0.05, ^##^ 0.01 < *p* < 0.05, ^###^ *p* < 0.01, vs. pPG-N1-pBD2/*27-2*; *n* = 3, per group, ns: not significant (*p* ≥ 0.05)).

The infiltration of neutrophils, macrophages, and eosinophils is reflected in the levels of MPO, NAG, and EPO, respectively. In colonic tissues, the DSS group dramatically raised MPO and EPO activities while lowering NAG activity as compared to the PBS group. Oral administration of pPG-PPT/*27-2* partially reversed these changes, whereas pPG-N1-pBD2/*27-2* treatment exerted a more pronounced effect by significantly suppressing the DSS-induced increases in MPO and EPO activities and restoring NAG activity. These results indicate that the pPG-N1-pBD2/*27-2* effectively alleviates DSS-induced intestinal inflammation by modulating inflammatory cell infiltration (Figure 7a).

The iNOS and COX-2 expression levels are crucial markers for assessing the degree of colitis and the effectiveness of anti-inflammatory treatments. Compared with the PBS control group, DSS treatment significantly increased the levels of iNOS and COX-2 in colonic tissues. The pPG-PPT/*27-2* partially reduced these inflammatory enzyme levels, whereas treatment with pPG-N1-pBD2/*27-2* produced a more pronounced effect (Figure 7b). These findings indicate that pPG-N1-pBD2/*27-2* effectively modulates inflammation-related factors and alleviates DSS-induced intestinal inflammatory responses.

Following DSS-induced colitis, serum cytokine levels were quantified by ELISA. As shown in Figure 7c, the DSS group’s levels of TNF-α, IL-1β, IL-6, and IL-10 were noticeably higher than those of the control group. Oral administration of either pPG-N1-pBD2/*27-2* or pPG-PPT/*27-2*, on the other hand, selectively raised IL-10 levels while dramatically lowering blood levels of IL-1β, IL-6, and TNF-α in comparison to the DSS model group. Notably, these effects were more pronounced in mice treated with pPG-N1-pBD2/*27-2*. Collectively, these results indicate that pPG-N1-pBD2/*27-2* administration effectively modulates systemic inflammatory cytokine responses in DSS-induced colitis.

### 3.7. Effects of pPG-N1-pBD2/27-2 on Intestinal Immune Function in Mice

**Figure 8:**
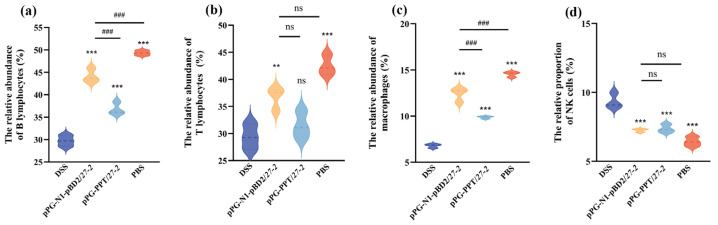
Proportions of immune cell populations in the spleens of mice from different groups. Proportions of B lymphocytes (**a**), T lymphocytes (**b**), macrophages (**c**), and NK cells (**d**) in the spleens of mice from different treatment groups (** 0.01 < *p* < 0.05, *** *p* < 0.01 vs. DSS; ^###^ *p* < 0.01 vs. pPG-N1-pBD2/*27-2*; *n* = 3, per group, ns, no significant difference). The proportions of immune cells were calculated as the percentage of total isolated live splenocytes. Flow cytometry analysis was performed on 3 independent spleen samples per group, which were randomly selected from 8 mice in each group.

The alterations in splenic immune cell proportions were evaluated by flow cytometric analysis (representative histograms and gating information are provided in Supplementary Figure S1). Relative to the DSS group, oral administration of the pPG-N1-pBD2/*27-2* and pPG-PPT/*27-2* partially restored these immune alterations. Specifically, both treatments noticeably increased the proportions of B lymphocytes and macrophages in the spleen (*p* < 0.01), with the effect being more noticeable in the pPG-N1-pBD2/27-2 group (Figure 8a,c). In addition, the ratio of splenic T lymphocytes was noticeably elevated in mice treated with pPG-N1-pBD2/*27-2* (*p* < 0.01), reaching levels comparable to those of the PBS group (*p* > 0.05), whereas the pPG-PPT/*27-2* group showed only a non-significant upward trend (Figure 8b).

Furthermore, both pPG-N1-pBD2/*27-2* significantly reduced the proportion of splenic NK cells compared with the DSS model group (*p* < 0.01), with a greater reduction observed in the pPG-N1-pBD2/*27-2* group (Figure 8d). Collectively, these findings demonstrate that oral administration of pPG-N1-pBD2/*27-2* effectively modulates splenic immune cell subsets and ameliorates DSS-induced immune dysregulation in mice.

## 4. Discussion

Defensins demonstrate significant antimicrobial activity and immunomodulatory functions, being essential to several biological processes, including cell migration and proliferation. For instance, hBD-3 promotes wound healing by stimulating fibroblast migration [20]. Within the intestinal microenvironment, pBD2 not only boosts the migration of intestinal epithelial cells, thereby decreasing the occurrence of DSS-induced colitis [21], but also expedites mucosal repair by regulating immune cell activity and cytokine release to facilitate inflammation resolution. Several studies suggest that administering exogenous antimicrobial peptides through live *L. paracasei 27-2* carriers can expand the antibacterial spectrum [22] and improve intestinal barrier function [23]. This study used *Lactobacillus* as the host strain to increase the efficacy of pBD2, which has been demonstrated to achieve considerable intestine colonization after seven days of oral treatment [24]. We successfully constructed the pPG-N1-pBD2/*27-2*, which exhibited efficient secretion and expression of pBD2. The growth curve, acid tolerance, and bile salt tolerance of this strain were comparable to those of the parental strain, suggesting that the introduction of exogenous genes did not negatively impact its essential biological characteristics. This observation is critical for its potential use as an oral delivery vector. Additionally, Western blot analysis and indirect immunofluorescence confirmed that pPG-N1-pBD2/*27-2* could stably express the exogenous protein pBD2 and efficiently secrete it extracellularly, thereby enhancing the protein’s biological functions within the intestinal environment. As a key intestinal innate immune antimicrobial peptide, pBD2 possesses unique biological properties, including anti-pathogen activity, mucosal barrier protection and epithelial repair ability. Efficient secretion pattern in this study greatly facilitates pBD2 to directly exert its biological functions in the intestinal lumen microenvironment.

To evaluate functional verification, cytotoxicity assays were conducted to exclude the non-specific cytotoxic effects of the pPG-N1-pBD2/*27-2* secretory protein on IPEC-J2. The pBD2 showed no cytotoxicity to IPEC-J2 cells within 12.5–200 ng/mL and significantly promoted cell proliferation with the optimal effect at 100 ng/mL. Furthermore, cell scratch assays demonstrated that pBD2 markedly accelerated migration and repair mechanisms in injured locations, demonstrating its crucial function in encouraging intestinal epithelial cell renewal and aiding in mucosal repair. These data collectively suggest that pBD2 derived from recombinant *L. paracasei 27-2* possesses excellent biological activity, and it plays an important role in maintaining intestinal epithelial homeostasis, accelerating epithelial renewal and alleviating intestinal mucosal injury.

Colitis is a prevalent inflammatory disease in animal production that compromises intestinal barrier function and adversely affects animal growth performance. The DSS-induced colitis model is widely used in intestinal inflammation research due to its simplicity, stability, and high reproducibility [25]. The traditional markers for determining the prevalence of colitis are the DAI scores [26]. We employed a DSS-induced mouse colitis model to assess the therapeutic efficacy of pPG-N1-pBD2/*27-2*. Significant colon shortening and elevated DAI scores demonstrated that DSS therapy successfully produced colitis. In contrast, oral administration of the recombinant strain markedly alleviated these symptoms. To clarify the anti-inflammatory mechanism even more, we analyzed the activities of MPO, EPO, and NAG, which are key markers for neutrophil, eosinophil, and macrophage infiltration, respectively [27,28]. Consistent with prior research, DSS induction resulted in elevated MPO and EPO activities, accompanied by a reduction in NAG activity, indicating substantial inflammatory infiltration and immune disruption. Notably, treatment with pPG-N1-pBD2/*27-2* reversed these trends by decreasing MPO and EPO levels while restoring NAG activity. These findings suggest that the recombinant strain may alleviate colitis, which is associated with suppressed aberrant inflammatory cell infiltration and improved colonic immune microenvironment.

Histopathological assessment revealed severe damage to the colonic mucosa in the DSS model group, characterized by extensive inflammatory cell infiltration and structural disarray, including the distortion or loss of glands and crypts. For intestinal homeostasis to be maintained, the intestinal epithelial barrier must remain intact [29]. This barrier is composed of multiple elements, including intestinal epithelial cells, the mucus layer, and antimicrobial peptides. Crucially, the physical barrier function is primarily established by tight junctions connecting adjacent epithelial cells, a process mediated by specific TJPs [30,31]. Key proteins identified to date, such as TJPs, collectively maintain the structural stability of the epithelium [32]. Recent study suggests that by regulating the expression levels of these TJPs, defensins help maintain the integrity of the intestinal epithelial barrier [33]. In the present study, DSS treatment was found to significantly reduce the expression levels of the TJPs in the mouse colon. Conversely, these proteins were expressed more when pPG-PPT/*27-2* was administered. Notably, oral treatment with the pBD2-expressing pPG-N1-pBD2/*27-2* restored the levels of these TJPs to values comparable to those observed in the PBS control group. Collectively, these findings suggest that porcine-derived *L. paracasei 27-2* and pBD2 are associated with the improvement of DSS-induced intestinal mucosal barrier injury, along with elevated expression of tight junction proteins, although barrier protection was primarily inferred from marker expression rather than direct functional permeability testing.

Previous studies have established that nitric oxide (NO) can upregulate the expression of COX-2, while COX-2 metabolites, in turn, induce the expression of iNOS. This reciprocal interaction creates a positive feedback loop that amplifies the inflammatory cascade and exacerbates tissue injury [34,35]. Furthermore, the dynamic equilibrium between pro-inflammatory and anti-inflammatory cytokines is fundamental to immune regulation, playing a pivotal role in maintaining host homeostasis and defense against infection [35]. Pro-inflammatory cytokines drive immune activation by promoting cell recruitment and antimicrobial activity [36], whereas IL-10 acts as a crucial negative regulator that limits excessive inflammation, protects tissues, and fosters immune tolerance [37,38]. For immunological homeostasis to be maintained, these mediators must be balanced in a coordinated manner. In this study, oral administration of the pPG-N1-pBD2/*27-2* caused TNF-α, IL-1β, and IL-6 levels to drop considerably, alongside a marked increase in IL-10 expression, compared with the DSS and the pPG-PPT/*27-2* groups. These findings suggest that the pPG-N1-pBD2/*27-2* strain possesses potential intestinal colonization ability, and it may alleviate DSS-induced colitis via regulating inflammatory responses.

While the present study did not directly quantify lamina propria lymphocyte recruitment, which would provide a more localized perspective, the examination of key inflammatory markers within colonic tissues, including MPO, NAG, EPO, iNOS, and COX-2, provides a reliable indirect representation of the characteristic features of the local intestinal immune response. These findings, alongside the observed histological cellular infiltration, constitute a robust evidence chain that effectively mirrors the core features of the local immune response.

The initial line of protection for the host is innate immunity, employing macrophages and NK cells to rapidly eliminate pathogens and trigger initial immune responses [39]. In contrast, adaptive immunity is characterized by high specificity; it amplifies immune responses through the coordinated action of T and B lymphocytes, which facilitates antibody production and targeted defense [40,41]. Collectively, these two systems work synergistically to establish a comprehensive defense network and maintain internal homeostasis [42]. The present study showed that the ratios of splenic macrophages, T lymphocytes, and B lymphocytes decreased considerably in DSS-induced colitis, whereas the fraction of NK cells was abnormally increased. The initial insult caused by DSS results in the disruption of the intestinal epithelial barrier, as evidenced by the downregulation of tight junction proteins [43]. This disruption facilitates the translocation of luminal antigens and the subsequent release of potent inflammatory mediators, such as CCL5 [44]. The spleen is an important secondary lymphoid organ involved in systemic immune surveillance and leukocyte trafficking [45]. During inflammatory stress, dynamic changes in splenic immune cell composition may reflect systemic immune activation and redistribution processes. Rather than indicating an overall compromise of immune function, alterations in the proportional representation of immune subsets may signify immune adaptation to inflammatory challenge. In the context of DSS-induced colitis, disruption of the intestinal barrier can trigger systemic inflammatory signaling, potentially influencing splenic immune cell dynamics. It is important to note that the present study evaluated the proportions of splenic immune subsets rather than absolute cell counts. Therefore, the observed reductions in certain populations may reflect relative shifts in cellular composition rather than true depletion of immune cells.

DSS treatment was associated with systemic immune perturbation and altered immune subset distribution. Notably, pPG-N1-pBD2/*27-2* treatment modulated the relative proportions of splenic immune cell subsets. Specifically, treatment increased the proportions of macrophages and T and B lymphocytes while normalizing elevated NK cell levels toward those observed in control mice. Furthermore, the immunomodulatory effects of pPG-N1-pBD2/*27-2* appeared more pronounced than those of pPG-PPT/*27-2*.

Collectively, these findings suggest that oral administration of recombinant *L. paracasei 27-2* contributes to the regulation of systemic immune homeostasis, promoting a more balanced coordination between innate and adaptive immune responses during DSS-induced colitis. Notably, these findings reflect relative proportional shifts rather than absolute cell counts; thus, they suggest a modulation of the splenic immune profile rather than a definitive restoration of systemic homeostasis. Further studies including lamina propria immune phenotyping and splenic absolute cell quantification will be necessary to clarify the precise mechanisms underlying these systemic immune alterations.

While the current study highlights the immunomodulatory and barrier-protective effects of pBD2, the specific alterations in the murine intestinal microbiota remain to be fully elucidated. As an endogenous broad-spectrum antimicrobial peptide, pBD2 selectively inhibits intestinal pathogenic and opportunistic bacteria but rarely affects beneficial commensal bacteria and probiotics. Oral recombinant *L. paracasei* can colonize the intestinal tract and continuously secrete pBD2, thereby optimizing intestinal microbial structure, reducing harmful bacteria, maintaining microecological homeostasis, and further enhancing intestinal mucosal barrier integrity. Consistently, a recent study revealed that synergistic probiotic combination alleviates *Salmonella typhimurium*-induced intestinal injury via regulating immunity, gut barrier homeostasis and metabolic profiles using transcriptomic and metabolomic analyses [46]. Accordingly, intestinal microbiota regulation is an important potential mechanism for the intestinal protective effects of this engineered strain, which also represents a promising direction for future research. However, we acknowledge that the current in vitro and in vivo analyses were conducted with a sample size of *n* = 3. Furthermore, the histopathological evaluation in this study relied on qualitative descriptive analysis rather than a formal blinded scoring system. Due to the extensive utilization of tissue samples for multiple molecular assays, a numerical reevaluation was not feasible at this stage. While this was sufficient to demonstrate statistical significance and high consistency among biological replicates in this study, it remains the minimum requirement for robust statistical comparison.

Subsequent research will employ metagenomic evaluation to systematically characterize changes in microbiota diversity and community structure, using a larger cohort and standardized quantitative scoring to further validate the robustness of these findings and to more comprehensively capture biological variability. Overall, the recombinant *L. paracasei 27-2* demonstrates significant potential as a feed additive and immune adjuvant due to its enhanced stability and immunogenicity. Future work will focus on evaluating the prolonged administration of this strain to assess the impact of sustained pBD2 production and rule out any potential side effects on host health, contingent upon rigorous safety and efficacy evaluations in animal models before developing optimal application forms.

## 5. Conclusions

In conclusion, our research demonstrates that the pBD2-secreting recombinant *L. paracasei* pPG-N1-pBD2/*27-2* significantly mitigates DSS-induced colitis in murine models. This effect is achieved by strengthening the intestinal mucosal barrier through the upregulation of tight junction proteins and maintenance of mucosal architecture. Additionally, it reestablishes the balance of the innate–adaptive immune axis by inhibiting the iNOS/COX-2 pathway, modulating cytokine levels, and restoring systemic immune cell homeostasis. Collectively, our findings provide important insights into IBD therapy and lay a solid foundation for subsequent related research.

## Figures and Tables

**Table 1:** Experimental mice groups.

Group	Oral Dose	Number
PBS	200 μL	8
pPG-N1-pBD2/*27-2*	200 μL (2 × 10^9^ CFU)	8
pPG-PPT/*27-2*	200 μL (2 × 10^9^ CFU)	8
DSS	200 μL	8

**Table 2:** Standard for evaluation of the DAI.

Weight Loss (%)	Occult Blood/Bloody	Fecal Property	Score
0	Occult blood negative	Normal	0
1–5	Occult blood negative	Loose	1
6–10	Positive for occult blood	Loose	2
11–15	Positive for occult blood	Loose stools	3
>15	Naked-eye bloody stool	Loose stools	4

**Table 3:** Identification of pBD2 expressed of the pPG-N1-pBD2/*27-2*.

Incubation Time	Supernatant (μg/mL)	Precipitation (μg/mL)
6 h	0.27 ± 0.02	0.47 ± 0.02
10 h	0.32 ± 0.04	0.78 ± 0.04
14 h	0.54 ± 0.06	1.65 ± 0.03
18 h	0.64 ± 0.05	2.21 ± 0.05
22 h	0.59 ± 0.06	1.11 ± 0.09
24 h	0.50 ± 0.03	0.69 ± 0.03

Note: Values are expressed as mean ± SD (*n* = 3), and values in blue represent the maximum.

**Table 4:** Viability of the pPG-N1-pBD2/*27-2* under different pH conditions.

Strains	Survival (%)			
	pH = 2	pH = 3	pH = 4	pH = 5
pPG-N1-pBD2/*27-2*	1.60 ± 0.14 ^a^	10.74 ± 1.38 ^a^	31.35 ± 2.06 ^a^	42.77 ± 1.86 ^a^
pPG-PPT/*27-2*	1.56 ± 0.12 ^a^	11.56 ± 1.11 ^a^	31.29 ± 2.11 ^a^	41.25 ± 2.01 ^a^

**Table 5:** Viability of the recombinant *L. paracasei 27-2* under different concentration of bile salt.

Strains	Survival (%)	
	0.1% Bile Salt Concentration	0.5% Bile Salt Concentration
pPG-N1-pBD2/*27-2*	2.64 ± 0.19 ^a^	0.24 ± 0.12 ^a^
pPG-PPT/*27-2*	2.56 ± 0.22 ^a^	0.16 ± 0.06 ^a^

Note: Bacterial viability was calculated relative to recombinant *L. paracasei* cultured under normal culture conditions and expressed as percentage (%). Values are expressed as mean ± standard deviation (SD) (*n* = 3). Means sharing the same letter are not significantly different (pPG-N1-pBD2/*27-2* vs. pPG-PPT/*27-2*, *p* > 0.05).

## Data Availability

The data that support the findings of this study are openly available in Mendeley Data at http://doi.org/10.17632/p6mw8cwc8f.1.

## Supplementary Materials

The following supporting information can be downloaded at: https://www.mdpi.com/article/10.3390/ani16101425/s1, Figure S1: Primer sequence; Table S1: Proportions of immune cell populations in the spleens of mice from different groups.

## Abbreviations

The following abbreviations are used in this manuscript:

pBD2	Porcine β-defensin 2
IFN-γ	Interferon-γ
IL-2	Interleukin-2
IL-10	Interleukin-10
MRS	Man Rogosa and Sharpe
PBS	Phosphate-buffered saline
SDS	Sodium dodecyl sulfate
PVDF	Polyvinylidene fluoride
HRP	Horseradish peroxidase
FITC	Fluorescein isothiocyanate
DAPI	4′,6′-diamino-2-phenylindole
ELISA	Enzyme-linked immunosorbent assay
IL-4	Interleukin-4
IPEC-J2	Immortalized Porcine Enterocyte Cell line J2
TCA	Trichloroacetic acid
DAI	Disease activity index
DSS	Dextran sulfate sodium
IBD	Inflammatory bowel disease
