# Supplementary material for: Protective Effects of Recombinant Lactobacillus paracasei Expressing Porcine β-Defensin 2 Against DSS-Induced Colitis in a Murine Model
Source: Animals (Basel). 2026 May 7;16(10):1425. doi: 10.3390/ani16101425 (PMC13203280; doi:10.3390/ani16101425)
Supplement: Supplementary file 1 [file animals-16-01425-s001.zip › Supplementary Table S1.pdf]

Table S1. Primer sequence

Primer	Primer Sequence (5′→3′)	Target Gene
ZO-1-F	TCATCCCAAATAAGAACAGAGC	ZO-1
ZO-1-R	AACAAAAAAATTAG	
Claudin-2-F	GCTGGGTTTCATCCTGGCTTCT	Claudin-2
Claudin-2-R	CCTGAGCGGTCACGATGTTGTC	
Occludin-F	ACTGGGTCAGGGAATATCCA	Occludin
Occludin-R	TCAGCAGCAGCCATGTACTC	
β-actin-F	TTCCTTCTTGGGTATGGAAT	β-actin
β-actin-R	GAGCAATGATCTTGATCTTC	
