# Supplementary figures and images for: Protective Effects of Recombinant Lactobacillus paracasei Expressing Porcine β-Defensin 2 Against DSS-Induced Colitis in a Murine Model
Source: Animals (Basel). 2026 May 7;16(10):1425. doi: 10.3390/ani16101425 (PMC13203280; doi:10.3390/ani16101425)

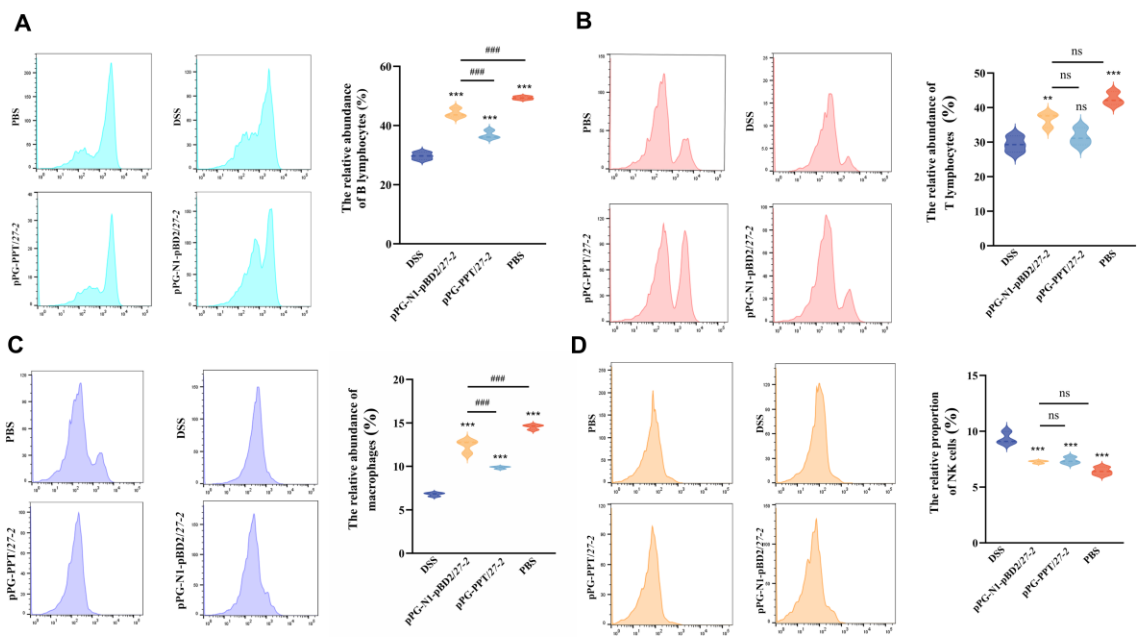

Figure S1. Primer sequence.

Supplement: Supplementary file 1 [file animals-16-01425-s001.zip › Supplementary Figure S1.pdf]
